# Prominent Vessel Sign on Susceptibility-Weighted Imaging in Acute Stroke: Prediction of Infarct Growth and Clinical Outcome

**DOI:** 10.1371/journal.pone.0131118

**Published:** 2015-06-25

**Authors:** Chia-Yuen Chen, Chin-I Chen, Fong Y. Tsai, Ping-Huei Tsai, Wing P. Chan

**Affiliations:** 1 Department of Radiology, Wan Fang Hospital, Taipei Medical University, Taipei, Taiwan; 2 Department of Radiology, School of Medicine, College of Medicine, Taipei Medical University, Taipei, Taiwan; 3 Department of Neurology, Wan Fang Hospital, Taipei Medical University, Taipei, Taiwan; 4 Department of Radiological Sciences, University of California Irvine, Irvine California, United States of America; 5 Imaging Research Center, Taipei Medical University, Taipei, Taiwan; 6 Department of Medical Imaging, Taipei Medical University Hospital, Taipei Medical University, Taipei, Taiwan; Henry Ford Health System, UNITED STATES

## Abstract

**Background and Purpose:**

Predicting the risk of further infarct growth in stroke patients is critical to therapeutic decision making. We aimed to predict early infarct growth and clinical outcome from prominent vessel sign (PVS) identified on the first susceptibility-weighted image (SWI) after acute stroke.

**Materials and Methods:**

Twenty-two patients with middle cerebral artery (MCA) infarction had diffusion-weighted imaging, SWI, MR angiography, and clinical evaluation using the National Institutes of Health Stroke Scale at 7–60 hours and 5–14 days after stroke onset. Late-stage clinical evaluation at 1 and 3 months used the modified Rankin Scale. The infarct area and growth were scored from 10 (none) to 0 (infarct or growth in all 10 zones) using the Alberta Stroke Program Early CT Score (ASPECTS) system.

**Results:**

Infarct growth on the second MRI occurred in 13 of 15 patients with PVS on the first MRI and not in any patient without PVS (n=7; *r*=0.86, *P*<0.001). The extent of PVS was significantly correlated with infarct growth (*r*=0.82, *P*<0.001) and early-stage outcome (*P*=0.02). No between-group difference in late-stage clinical outcome was found.

**Conclusion:**

PVS on the first SWI after acute MCA territory stroke is a useful predictor of early infarct growth. Extensive PVS within the large MCA territory is related to poor early-stage outcome and could be useful for clinical assessment of stroke.

## Introduction

Predicting the risk of further infarct growth in stroke patients is critical to therapeutic decision making [1]. Because ischemic stroke follows a dynamic course during the first few days or weeks, serial diffusion-weighted imaging (DWI) is better than measurement at a single time point for predicting clinical outcome [2,3]. Early infarct growth shown in serial images within 5–7 days is an independent predictor of poor outcome [4]. However, serial DWI is time-consuming and is not feasible for some patients.

Perfusion-weighted imaging (PWI) reflects cerebral blood flow and cerebral blood volume. The mismatch between PWI and DWI predicts a favorable response to thrombolysis after early reperfusion, and so may be a surrogate for ischemic penumbra [1,5], although its value for predicting infarct growth or clinical outcome is controversial and has been challenged [1,6,7]. In addition, PWI requires administration of contrast agent, which limits its application in patients with renal insufficiency.

T2*-sensitive MRI or susceptibility-weighted imaging (SWI) is a potential alternative for predicting infarct growth. In the ischemic brain, the increased oxygen extraction fraction and slow flow contribute to a higher level of deoxyhemoglobin and vein dilatation, which increases the conspicuity of vessels on SWI [8–11]. This prominent vessel sign (PVS) on SWI has been reported to indicate increased oxygen extraction and correlates well with venous and capillary deoxyhemoglobin levels [12–17]. The area may be a target for acute therapy [18]. Kaya et al. [8] identified multiple hypointense vessels strictly in the ischemic territory during the hyperacute phase of stroke on gradient T2* at 3T. The region was larger than the lesion shown on DWI and correlated well with the final infarction area after 72 hours. Haacke et al. [19] used SWI on a higher-resolution 1.5 T machine, which depicts and enhances magnetic susceptibility effects better than the conventional T2* gradient sequence. SWI/DWI mismatch has also been recommended as a potential indicator of infarct growth in some reports [14,20,21]. Recently, two studies on pediatric arterial ischemic stroke have reported that SWI/DWI mismatch [22] and SWI/diffusion tensor imaging mismatch [23] showed middle cerebral artery (MCA) infract progression on follow-up images. Using a similar approach, in our study, we aim to investigate whether the risk of early infarct growth could be predicted by PVS on a single SWI in adult patients. We hypothesized that (when reperfusion is not established in time) patients with extensive PVS would have a larger infarct growth and higher incidence of early neurological deterioration.

## Materials and Methods

The Institutional Review Board of Taipei Medical University-Wanfang Hospital approved this prospective study and participants provided their signed informed consents.

### Subjects

From July 2009 to December 2012, we prospectively evaluated patients with acute brain infarct in the territory of the MCA consecutively presented to our emergency care unit. All patients received routine neurological examination, MRI scanning, and standard medical treatment. We excluded patients with parenchymal hemorrhage on the first image, MRI more than 72 hours after stroke onset, early death, and those who declined to join the study. Initially, 39 patients were recruited to this study. Follow-up MRI, including DWI and SWI, was performed again at least 7 days later when the patient’s condition had stabilized or earlier if the clinical condition worsened. Of these 39, 17 patients were excluded because of parenchymal hemorrhage (n = 4), clinical evidence of systemic emboli formation (n = 7) and poor quality of MRI images (n = 6). Finally, 22 patients were included in the study (S1 Table).

### Clinical assessment

Three neurologists evaluated the clinical condition of all patients. We used the National Institutes of Health Stroke Scale (NIHSS) on the day the patient arrived for initial neurological evaluation and on the day of follow-up MRI for early-stage evaluation. For late-stage neurological evaluation, we used a modified Rankin Scale (mRS) the first month and the third month after stroke onset.

Patients were divided into the good-improvement group (i.e., those with a second NIHSS score ≤2 or improvement between the first and second NIHSS score ≥4) and the poor-improvement group (all others).

### MRI protocol

All the MRI studies were performed at our institution on a 1.5 T scanner (Magnetom Avanto; Siemens Medical Solutions, Erlangen, Germany) with a standard 12-channel head coil. After the routine T1-weighted and T2-weighted fluid attenuation inversion recovery (FLAIR) sequence, DWI and SWI sequences were performed to characterize the ischemic regions. For the DWI sequences, we used single-shot echo-planar imaging (with TR/TE = 3700/109 ms, b = 1000 s/mm^2^, slice thickness = 5 mm, slice number = 28, and matrix = 128x128) and generated ADC maps. For the transverse 3-dimensional (3D) SWI sequences, we used TR/TE = 49/40 ms, flip angle = 15°, slice thickness = 2 mm with 60 sections per slab, matrix = 224×256, 64 slices, and (integrated parallel acquisition technique (iPAT) acceleration factor = 2. Minimal intensity projection (minIP) images were reconstructed with an effective minIP thickness of 16mm. The sequence, along with all image processing, was automated on the Siemens MR scanner platform based on the concepts of Haacke et al. [19]. The phase, magnitude (mag), minIP, and SWI images were uploaded and made available on a picture archiving and communication (PACS) system (IMPAX 6.4; AGFA Healthcare, Mortsel, Belgium). To determine the location of the arterial occlusion, magnetic resonance angiography with a 3D time-of-flight sequence was performed (with TR/TE = 25/7ms, flip angle = 25°, field of view = 20×16 cm, matrix = 256×256, 3 slabs, 44 slices per slab, and slice thickness = 0.8 mm). The total scan time for all protocols was less than 30 minutes.

### Analysis of MRI images

All MRI images were interpreted by consensus of two radiologists experienced in neuroimaging (C.Y.C; W.P.C). All initial MR images were interpreted blind to the clinical findings of each patient. The area of infarct growth on the follow-up MRI was compared with the acute infarct area on the initial MRI with blinding. The definition of an acute infarct area was high signal intensity on DWI with dark signal intensity on ADC. The infarct extent was scored using the Alberta Stroke Program Early CT Score (ASPECTS) system, a 10-point semiquantitative CT score system developed and tested as a reliable grading system to assess the extent of ischemic change and to predict functional outcome in patients with acute ischemic stroke [11]. This topographic system allots 1 point for each of 10 zones of the MCA territory. A score of 10 is normal while 0 indicates diffuse infarction [24]. The application of ASPECTS to DWI in stroke has been extended and contributes to outcome prediction and quick risk assessment before thrombolytic therapy [25].

Infarct growth was defined as any new or larger lesion on the second MRI. The location of infarct growth was recorded for the caudate nucleus, lentiform nucleus, internal capsule, insula, M1, M2, and M3 (anterior, middle, and posterior third of the lower MCA territory, respectively), and M4, M5, and M6 (anterior, middle, and posterior third of the higher MCA territory, respectively), based on the ASPECTS topographic system. The infarct growth was scored from 10 (no growth) to 0 (growth in all 10 zones).

Parenchymal hematoma, an exclusion criterion, was defined as blood clot signal intensity on T1-weighted and/or T2-weighted sequences, with mass effect. Microbleed, not an exclusion criterion, was defined as small dark signal dots (maximum diameter < 10 mm) on SWI.

**Fig 1:**
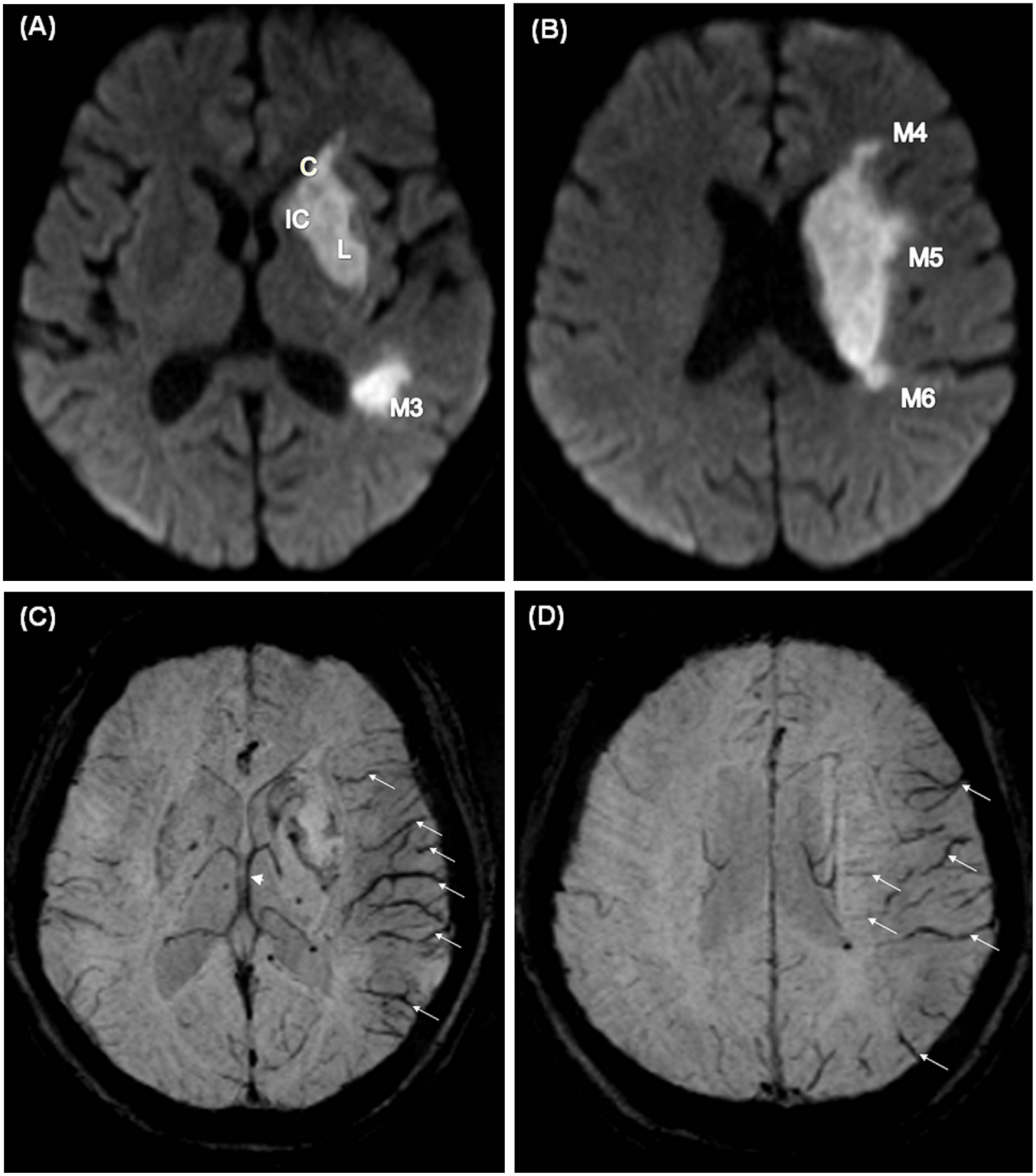
A 63-year-old woman had a diagnosis of left middle cerebral artery territory infarct. The infarct was noted in all 10 zones on diffusion-weighted or susceptibility-weighted magnetic resonance imaging with ASPECTS score = 0. Diffusion-weighted imaging **(A,B)** at the basal ganglion and suprabasal ganglion levels reveals the infarct in the internal capsule, lentiform nucleus, caudate nucleus, and zones M3, M4, and M5 of the middle cerebral artery. Susceptibility-weighted imaging reveals prominent hypointense cortical and medullary vessels diffusely seen in the insula and M1 to M6 zones of the left middle cerebral artery territory. Engorged deep veins and thalamostriate artery over the lesion side compared with the healthy side were also noted. Involved M1 to M6 zones and insula lost 7 points and an engorged thalamostriate vein lost 3 points. The prominent vessel sign score was 0 (10–7–3 = 0). Susceptibility-weighted imaging **(C, D)** at the basal ganglion and suprabasal ganglion levels reveals prominent vessel signs in the cortical veins (arrows), medullary veins (arrows) and thalamostriate vein (arrowhead).

**Fig 2:**
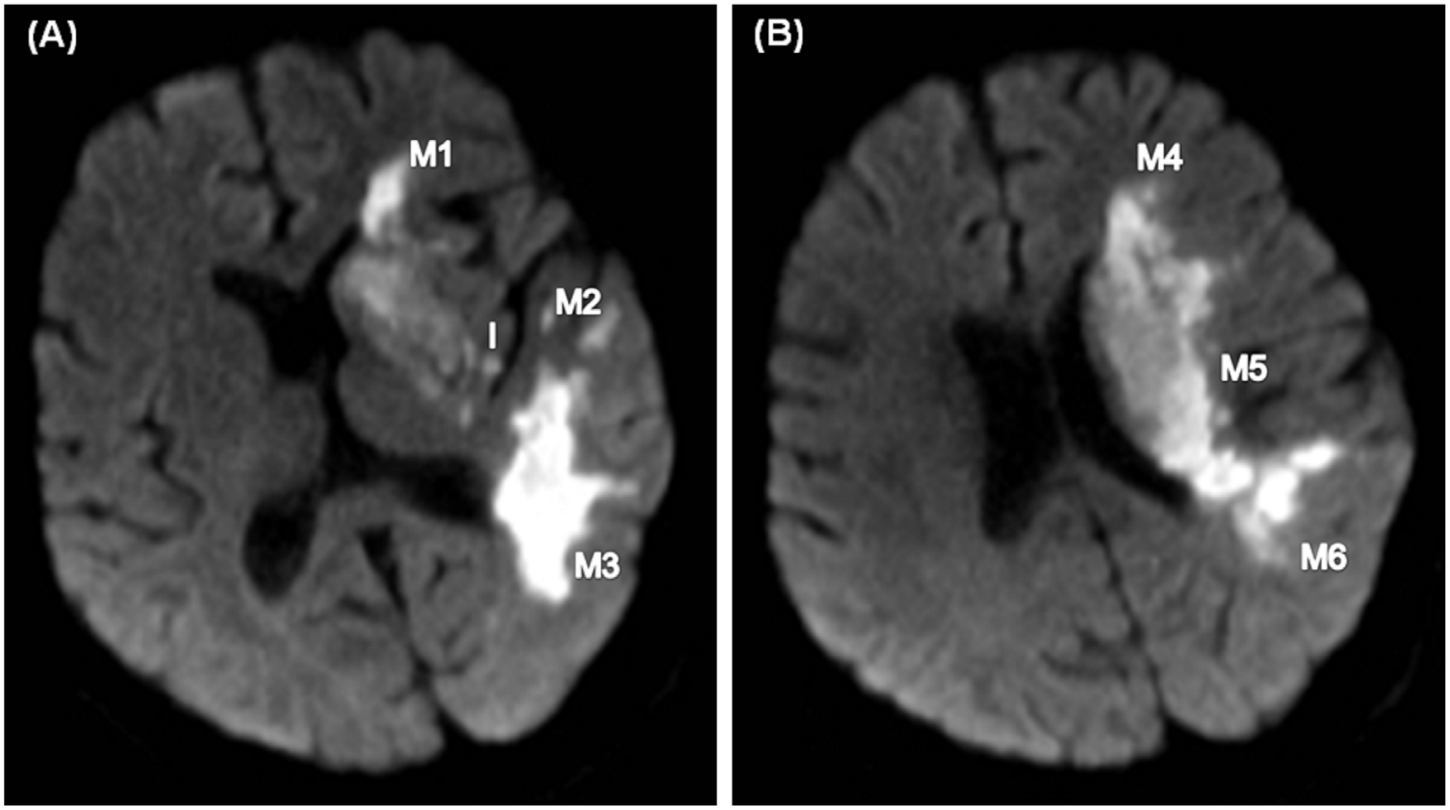
Same patient as in Fig 1 The second diffusion-weighted imaging study reveals new or larger infarct areas in the internal capsule, insula, lentiform nuclei, and zones M1 to M6. The infarct growth score was 1 (10–3–6 = 1). The second diffusion-weighted image (**A, B**) at the basal ganglion and suprabasal ganglion levels reveals infarct growth in the insula and zones M1 to M6.

The PVS on SWI was defined as a local prominence of hypointense vessels with either increased vessel number or diameter in the target area, when compared with the non-target area. In this study, the target area was defined as the MCA territory of the infarct side. For correlation with infarct growth, the same topographic system was used to record the extent of the PVS. The PVS of the insular cortical vessels was recorded as I of the lower MCA-territory cortical or medullary vessels (M1, M2, or M3), of the higher MCA-territory cortical or medullary vessels (M4, M5, or M6), and of the thalamostriate vein (C, L, or IC) because this vein drains the caudate nucleus, lentiform nucleus, and internal capsule. After the two readers had reached a consensus, the extent of the PVS was scored from 10 (no PVS) to 0 (PVS in M1, M2, M3, M4, M5, M6, I, C, L, or IC). An example is shown in Figs 1 and 2.

An intra-arterial blood clot on SWI was defined as a focal hypointensity within the intracranial arteries, with the diameter of hypointensity exceeding the parent vessel diameter. Arterial occlusion was defined as loss of high signal intensity in the artery lumen and nonopacification of distal parts on MRA.

### Statistical analysis

Statistical analysis used SPSS, version 17.0 (SPSS, Chicago, IL, USA). Mean and standard deviation of PVS scores, DWI ASPECTS scores, and infarct growth scores were calculated. Spearman’s rank correlation test was used to examine the correlations between PVS score and infarct growth score, change in NIHSS scores, first-month mRS score, and third-month mRS score. Regression was computed, with *P*<0.05 considered significant.

We divided patients into the P (positive) group (PVS score < cutoff point) and N (negative) group (PVS score ≥ cutoff point). We used PVS = 10, 9, 8, 7, 6, 5, 4, 3, 2, 1, and 0, respectively, as cutoff points. Patients were also divided into two subgroups according to other image data including arterial occlusion on MRA, intra-arterial clot, microbleed, and initial infarct ASPECTS scores (ASPECTS score = 9, 8, 7, 6, 5, 4, 3, 2, 1, 0 as cutoff points), respectively. Correlation between image data and clinical data (NIHSS scores, the first-month mRS score, and third-month mRS score) was examined by McNemar’s test. Cramer’s V coefficients were calculated to compare contingencies.

## Results

The study included 10 women and 12 men, aged 44–85 years (mean 67.1 years). First MRI images were all acquired in the acute stage of stroke (mean 32.9 hours, range 7–60 hours) and second images at least 7 days after stroke for 19 patients and earlier for 3 patients (mean 10.3 days after stroke, range 5–14 days). The MCA territory infarct was on the right side in 9 patients and on the left in 13. The mean ASPECTS score was 4.3 (range 0–9). Arterial occlusion was detected in 19 patients, intra-arterial blood clots in 9, and microbleed in 6. PVS was detected in 15 patients (mean score 4.1, range 0–10).

The presence of PVS was significantly correlated with arterial occlusion (*P* = 0.006) and intra-arterial blood clot (*P* = 0.008), but poorly correlated with microbleed (*P*>0.5). The interval from stroke onset to first MRI scan was similar between patients with and those without PVS (Levene's test, *P* = 0.17).

**Fig 3:**
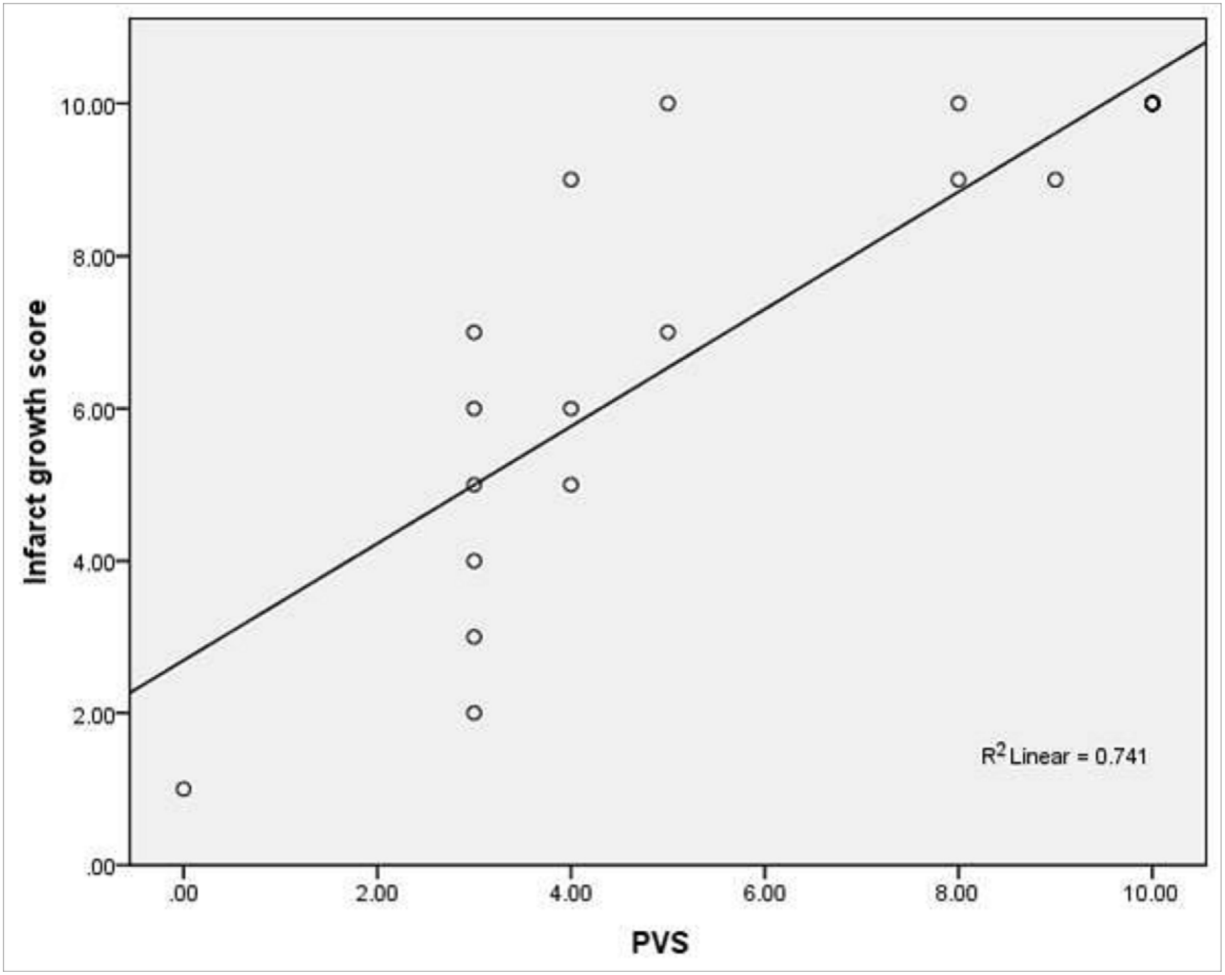
Correlation between prominent vessel sign (PVS) score on the first magnetic resonance image and infarct growth score (*n* = 22, *r* = 0.86, *P*<0.001).

The second MRI revealed no infarct growth in 9 patients and infarct growth in 13 (ASPECTS mean score 3.95, range 0–9; mean infarct growth score 7.4, range 0–10). Of 7 patients without PVS on the first MRI, none had infarct growth on the second. Of 15 patients with PVS on the first MRI, 13 (87%) had infarct growth. The PVS score and infarct growth score were well correlated (*r* = 0.86, *P*<0.001) (Fig 3). Extent of infarct growth on the second MRI matched the extent of PVS on the first MRI (Cramer’s *V* = 0.82, *P*<0.001) (Table 1). Our results were consistent with those of recently published studies on pediatric arterial ischemic stroke in which we restricted the analytic sample to adults, indicating SWI/DWI mismatch is useful for predicting ischemic stroke progression [23].

**Table 1:** Zonal distribution of prominent vessel sign on the first magnetic resonance imaging scan and presence of infarct growth on the second scan.

	Patients with PVS (*n* = 15)				Patients without PVS (*n* = 7)	
Territory	PVS(+) IG(+)	PVS(+) IG(–)	PVS(–) IG(+)	PVS(–) IG(–)	PVS(–) IG(+)	PVS(–) IG(–)
Caudate nucleus	0	1	3	11	0	7
Lentiform nucleus	1	0	3	11	0	7
Internal capsule	1	0	0	14	0	7
Insula	5	3	0	7	0	7
M1*	6	3	0	6	0	7
M2*	2	10	0	3	0	7
M3*	9	5	0	1	0	7
M4*	9	3	0	3	0	7
M5*	9	4	0	2	0	7
M6*	9	5	0	1	0	7

* Infarcts in M1 to M6 (6 zones of the territory of the middle cerebral artery) were estimated using the Alberta Stroke Program Early CT Score (ASPECTS) system.

PVS(+)/PVS(–), positive/negative prominent vessel sign on first MRI; IG(+)/IG(–), with/without infarct growth on second MRI.

The mean first NIHSS score was 11.3 (range 3–22) and the second 7.2 (range 1–26). Only a fair correlation was found between PVS scores and the change in NIHSS scores (*r* = –0.41). The two patients with PVS and no infarct growth showed improvement from a first NIHSS score of at least 4 to a second score of 1. The first- and third-month mean mRS scores were 2.8 (range 1–6) and 2.4 (range 0–6), respectively. Neither was significantly correlated with PVS score.

In the paired correlation of clinical and image data of the NIHSS subgroup, the correlation of NIHSS score with PVS was highest when PVS = 8 was the cutoff point. Only a weak or poor correlation was found for other variables. With PVS = 8 as a cutoff point, the P group (score <8) had 12 patients and N group (score ≥8) 10 patients. All 10 N-group patients had a good early-stage clinical outcome and 40% (5 of 12 patients) in the P group had poor early-stage clinical outcome. The correlation between the PVS and NIHSS score groups showed that PVS is a significant factor affecting NIHSS (*P* = 0.02) (Table 2).

**Table 2:** Correlation between prominent vessel sign and clinical outcomes.

Prominent vessel sign score	Good improvement (*n*)	Poor improvement (*n*)
<8 (P group)	7	5
≥8 (N group)	10	0

**Fig 4:**
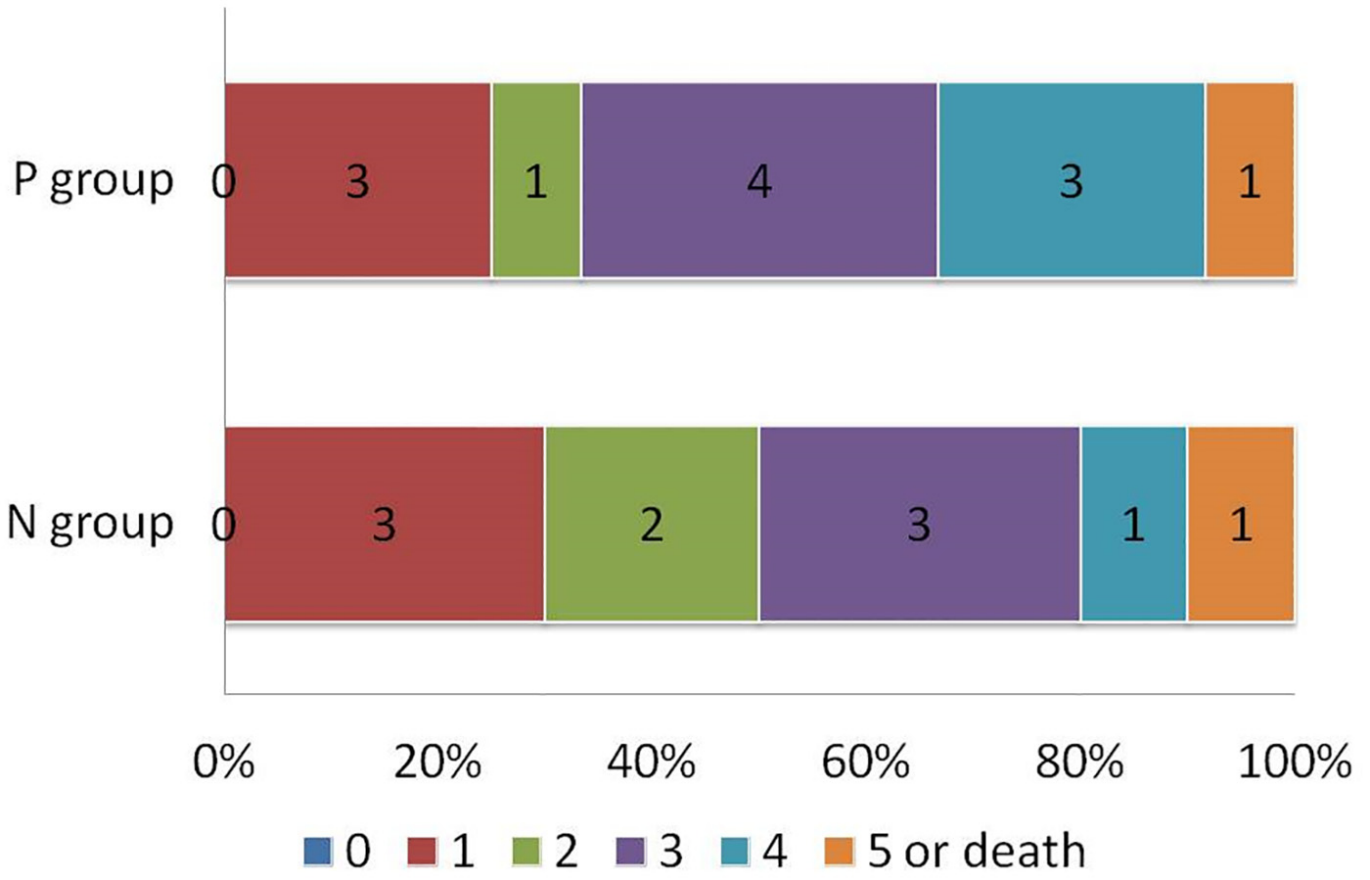
Late-stage outcome at 30 days according to the score on the modified Rankin Scale (*n* = 22, *r* = –0.22). P group (*n* = 12), with PVS score <8. N group (*n* = 10), with PVS score ≥8.

The PVS P group (compared with the PVS N group) tended to have poorer late-stage clinical outcome (as correlated with the first-month mRS), but this was not statistically significant (Fig 4).

## Discussion

Our study showed that the PVS on SWI is a signature of salvageable ischemic tissue that will become infarcted if blood perfusion cannot be established in time. This finding is consistent with the results of previous studies [14,20–23,26–29]. The PVS had a positive predictive rate of 87% and a negative predictive rate of 100%. Good spatial correlation between infarct growth and the extent of PVS was also observed. Of 57 zones of infarct growth, all 46 in the insula or M1–M6 zones of the MCA territory matched the extent of PVS, consistent with previous reports that PVS on SWI can predict stroke evolution and spatially correlate with DWI/PWI mismatch [3,14,17,20–23,30]. In superficial zones, PVS related to infarct growth had a sensitivity of 100%, specificity of 62%, positive predictive rate of 59%, and negative predictive rate of 100%. On the other hand, PVS had low sensitivity in predicting infarct growth in deep zones. Only two patients (25%) with infarct growth in the lentiform nucleus, internal capsule, or caudate nucleus had PVS, which can be explained by the admixed venous flow in the thalamostriate vein, which drains not only these structures, but also the thalamus. Regarding the two patients (patient no. 4 and patient 21) with PVS but no infarct growth, both had arterial occlusion at the M1 segment of the MCA on MRA and no intra-arterial clot on SWI. Both of them had no PVS on follow-up SWI. We presumed that these two patients might have chronic arterial stenosis with only a tiny blood clot formed in the initial stage, and that soon thereafter they developed collaterals and reperfusion even though no thrombolytic agent was given. Their clinical outcome was obviously improved from initial NIHSS score 11 and 6 to 1 on follow up, respectively. The findings were consistent with Hermier’s study [31] that good collaterals and recanalization correlated with better clinical outcome. Patient 4 had some hypointense cortical vessels on the initial SWI which turned hyperintense on the follow-up SWI. PVS might reflect not only veins but also small arteries with deoxyhemoglobin blood in the penumbra area.

Consistent with previous SWI studies [26–29,32], our study of 22 patients showed PVS in 15, microbleed in 6, and intra-arterial thrombus in 9. A lower microbleed rate would be expected, with parenchymal hemorrhage used as an exclusion criterion. A previous study showed that T2*-weighted sequences can detect more intra-arterial thrombus, with a sensitivity of 83% and specificity of 100% [33]. This could be due to the susceptibility of SWI to artifacts near the skull base, which blur the visibility of Willis’s circle. As correlated MRA and SWI findings, we found that nine of 19 patients with arterial occlusion on MRA had intra-arterial blood clot on SWI. In three of these patients, the blood clot was distal to the occlusion (M1-2 segment versus ICA/MCA) and in six patients, location of the blood clot was well correlated with that of the arterial occlusion. That might reflect the various causes of arterial occlusion, with blood clot formation being only one of these causes.

Few SWI studies have reported clinical outcomes. In a retrospective study, Huang *et al*. [26] reported no correlation between PVS and clinical outcomes, including NIHSS change, hemorrhagic transformation, and brain edema. PVS was defined by comparison with control images and not quantified. In Baik’s study [27], clinical outcome improved with the apparent normalization of PVSs in veins after successful recanalization. One case report in the literature described that SWI iso- or hyperintensity of the draining veins might suggest hyperperfusion, which contributed further to an increased risk of developing post-ischemic malignant edema [34]. In Polan’s study [23], however, SWI-hyperintense venous signal on acute ischemic stroke did not predict the development of malignant edema. In our study, the correlation between PVS and the change in NIHSS score (*r* = –0.41) was only fair, and no significant correlation was found between PVS and mRS score. But if we used PVS = 8 as the cutoff point, the P group and N group had different early-stage clinical outcomes. Given that the extent of PVS indicates the extent of penumbra, that patients with more extensive PVS can be expected to have a larger volume of salvageable tissue to be rescued. These patients had greater likelihood of a larger area of infarct growth and early neurological deterioration: 5 of 12 patients in the P group had less improvement of NIHSS score or clinical deterioration, but all 10 patients in the N group had a good early-stage clinical outcome, with either more improvement of NIHSS score or limited neurological deficit. No PVS was seen in follow-up images, which could be due to the relationship of PVS to short-term infarct or to reperfusion. Also, the PVS P group tended to have poor first-month mRS scores, so it was not surprising that the correlation between PVS and three-month mRS was poor. PVS might be applicable only in the acute stage.

Various correlations between PVS and clinical outcome in different studies might reflect the effect of successful therapy rather than the volume of the penumbra. In our study, PVS correlated with worse early-stage clinical outcome in circumstances where only conservative treatment was provided. Eleven patients who had MRI beyond the approved time window for thrombolysis (5 patients at 12–24 hours and 6 patients at 24–48 hours) had PVS and infarct growth on follow-up. Evidence shows that potentially viable brain tissue may exist for up to 24 hours after the onset of ischemic stroke and in some cases up to 48 hours [35,36]. Theoretically, these patients might benefit from more aggressive therapy to rescue tissue at risk. However, Hermier’s T2*-weighted imaging study reported that PVS was a marker of high risk for hemorrhage when patients were treated with intravenous tissue plasminogen activator [37]. Whether patients with PVS would benefit from thrombolytic treatment needs further clarification.

The limitations of this study include the small patient number and potential for patient selection bias. Our MRI protocol did not include performing PWI or arterial spin labeling because we designed to reduce time and focused on SWI/DWI for patients with suspected acute stroke. Patients with the worst clinical outcomes or who died were not recruited because they usually had no follow-up MRI. Bias in interpreting the images was possible, because PVS in this study was defined by observation and comparison rather than objective measurement of vessel number or diameter. Using ASPECTS for PVS semiquantification is arguable. Quantitative susceptibility mapping is a development of SWI that utilizes phase data to obtain information on local susceptibility [38]. It may in future be possible to provide fully quantitative and noninvasive information on oxygen metabolism.

In conclusion, the PVS on SWI can predict MCA territory infarct growth, and the extent of infarct growth matches the extent of PVS. Extensive PVS within the large MCA territory correlates with poor early-stage clinical outcome, in contrast with a minimal or no PVS. PVS does not predict late-stage clinical outcome. SWI provides useful clinical information in acute ischemic stroke and potentially guides early treatment options to prevent infarct progression. Therefore, SWI should be added to the routine neuroimaging protocol for patients with suspected acute stroke.

## Supporting Information

S1 Table Clinical characteristics. (DOCX)
