# Supplementary material for: Prominent Vessel Sign on Susceptibility-Weighted Imaging in Acute Stroke: Prediction of Infarct Growth and Clinical Outcome
Source: PLoS One. 2015 Jun 25;10(6):e0131118. doi: 10.1371/journal.pone.0131118 (PMC4481350; doi:10.1371/journal.pone.0131118)
Supplement: S1 Table — (DOCX) [file pone.0131118.s001.docx]

**SI Table: Clinical characteristics***

*Infarcts in M1 to M6 (6 zones of the territory of the middle cerebral artery) were estimated using the Alberta Stroke Program Early CT Score (ASPECTS) system.

ASPECTS, Alberta Stroke Program Early CT Score; PVS, prominent vessel sign.
